# Predicting all‐cause mortality from basic physiology in the Framingham Heart Study

**DOI:** 10.1111/acel.12408

**Published:** 2015-10-08

**Authors:** William B. Zhang, Zachary Pincus

**Affiliations:** ^1^ Department of GeneticsWashington University in St. LouisSt. LouisMO63130USA; ^2^ Department of Developmental BiologyWashington University in St. LouisMO63130USA

**Keywords:** aging, biodemography, cumulative risk, mortality, physiology, risk prediction

## Abstract

Using longitudinal data from a cohort of 1349 participants in the Framingham Heart Study, we show that as early as 28–38 years of age, almost 10% of variation in future lifespan can be predicted from simple clinical parameters. Specifically, we found diastolic and systolic blood pressure, blood glucose, weight, and body mass index (BMI) to be relevant to lifespan. These and similar parameters have been well‐characterized as risk factors in the relatively narrow context of cardiovascular disease and mortality in middle to old age. In contrast, we demonstrate here that such measures can be used to predict all‐cause mortality from mid‐adulthood onward. Further, we find that different clinical measurements are predictive of lifespan in different age regimes. Specifically, blood pressure and BMI are predictive of all‐cause mortality from ages 35 to 60, while blood glucose is predictive from ages 57 to 73. Moreover, we find that several of these parameters are best considered as measures of a rate of ‘damage accrual’, such that total historical exposure, rather than current measurement values, is the most relevant risk factor (as with pack‐years of cigarette smoking). In short, we show that simple physiological measurements have broader lifespan‐predictive value than indicated by previous work and that incorporating information from multiple time points can significantly increase that predictive capacity. In general, our results apply equally to both men and women, although some differences exist.

## Introduction

Aging is a complex biological process with multiple contributing genetic pathways (Kenyon *et al*., 1993; Lakowski & Hekimi, 1996; Hansen *et al*., 2007; Lunetta *et al*., 2007; Pan *et al*., 2007; Willcox *et al*., 2008; Li *et al*., 2009; Imai & Guarente, 2014; Satoh & Imai, 2014), significant variation among even closely related organisms (Jones *et al*., 2014), and an ongoing debate about its root causes and origin (Kirkwood, 2005). At its core, however, aging is simply the statistical phenomenon of an increased probability of death over time. In addition, mortality risk does not increase at equal rates at all ages (Vaupel *et al*., 1998). Further, aging can vary significantly for different individuals at the same age. As a result, an individual's chronological age is a limited predictor of their actual mortality risk.

In order to develop improved prognostic capability for mortality, demographers have sought to identify biomarkers of aging, functionally defined as a biological parameter of an organism that either can better predict functional capability or mortality risk than chronological age (Baker & Sprott, 1988). This is motivated by an array of valuable applications, including improved actuarial modeling, biological investigations of the mechanisms of aging and therapeutics to slow its progress, and the clinical ability to better target interventions to at‐risk patients.

Driven by these goals, researchers in the 1970s and 1980s proposed a large number of candidate biomarkers of aging (Baker, 1975; Ludwig & Smoke, 1980; Ingram & Reynolds, 1986). Although these studies identified several putative biomarkers, many of the published multivariable composite scores were overfit—that is, overly tuned to a very specific dataset. Consequently, these composite scores did not have broad applicability, and later studies failed to validate the original findings (Baker & Sprott, 1988; Costa & McCrae, 1988; Dean & Morgan, 1988; Ingram, 1988; Wilson, 1988).

As a result, much subsequent work has focused on predicting specific disease processes instead of all‐cause mortality (Cook *et al*., 1995; Vasan *et al*., 2001; Sesso *et al*., 2008). Many physiological parameters, such as blood pressure (Kannel, 1996) and body mass index (BMI) (Eckel *et al*., 1998), have been well‐characterized as risk factors for cardiovascular disease and other specific morbidities. In general, these studies have focused on middle‐aged individuals and have emphasized risk over a limited term, often just 5‐ or 10‐year periods (e.g., Assmann *et al*., 2002). In addition, most of the work on both general biomarkers of aging and specific risk factors has been limited to the analysis of data from single time points.

More recently, Yashin and colleagues have sought to use longitudinal data to gain insight into the overall aging process and the relationship between physiological change over time and mortality risk (e.g., Yashin *et al*., 2013). In particular, modeling physiological change as a dynamic system allowed mortality risk to be successfully modeled as a function of the difference between an individuals' current physiological state and the ideal state for an individual of that age (Yashin *et al*., 2007; Arbeev *et al*., 2011). Related analyses classified individuals' likely lifespans according to trajectories of physiological indices (Yashin *et al*., 2006, 2010). Further, a landmark series of studies of the accumulation of age‐related functional deficits in individuals over time identified a snowball effect where individuals with physiological deficits are more likely to accumulate further deficits over time and are exponentially more likely to die (Mitnitski *et al*., 2005, 2006). Taken together, these studies indicate that the trajectory of an individuals' physiological state over time provides a rich context for understanding future mortality risk.

We therefore set out to address two very simple questions about lifespan prediction from longitudinal data. First, given an individuals' history of physiological measurements (blood pressure, height, weight, and similar), how early in life is there any indication of that individuals' ultimate lifespan? A very recent investigation showed that that aging is indeed detectable even in young adults (Belsky *et al*., 2015). Specifically, these investigators found that young adults who showed signs of accelerated physiological aging also experienced greater functional impairment. We now ask whether such early‐life aging has detectable impacts on eventual survival decades hence.

Second, this work seeks to determine whether data from multiple time points can be aggregated in any way to provide a clearer estimate of future lifespan? Many of the models presented in previous work (e.g. Mitnitski *et al.,* 2006; Yashin *et al.,* 2007; Arbeev *et al.,* 2011) use longitudinal data, but estimate mortality risk at a particular time only from measurements made at that time. This effectively assumes that mortality risks are a memory‐free Markov process. In this work, we test this assumption and determine whether physiological history is helpful in lifespan prediction.

Our investigation of longer term biomarkers of longevity was made possible by the continued progression of the Framingham Heart Study (FHS). With its large cohort, consistent longitudinal measurements of basic physiology, and excellent level of follow‐up in terms of rate and length, the FHS offers a unique opportunity to investigate the contributions of biomarkers from very young ages, as well as the evolution of biomarker effectiveness throughout the aging process. The FHS population also allows us to focus on a single longitudinal cohort of individuals, avoiding the possible confounding effects of using additional cross‐sectional data. In this work, we use the Framingham data to understand the proportion of variation in all‐cause mortality that can be predicted from basic clinical measurements. We study these effects from early‐ to mid‐adulthood, and examine how they evolve as a result of the aging process (Table 1).

**Table 1:** Summary statistics of the changing demographics of the surviving cohort over time

	Living subcohort size	Mean age (years)	% Males	% Females	Number of males	Number of females
Exam 01	1349	34.5 ± 2.2	47	53	631	718
Exam 02	1329	36.6 ± 2.2	47	53	620	709
Exam 03	1311	38.5 ± 2.2	47	53	611	700
Exam 04	1301	40.5 ± 2.2	46	54	604	697
Exam 05	1285	42.5 ± 2.2	46	54	597	688
Exam 06	1270	44.5 ± 2.2	46	54	587	683
Exam 07	1256	46.5 ± 2.2	46	54	582	674
Exam 08	1236	47.9 ± 2.2	46	54	573	663
Exam 09	1216	49.9 ± 2.2	46	54	562	654
Exam 10	1189	52.0 ± 2.2	46	54	547	642
Exam 11	1162	53.8 ± 2.2	46	54	535	627
Exam 12	1136	56.0 ± 2.2	46	54	527	609
Exam 13	1108	58.0 ± 2.2	46	54	512	596
Exam 14	1071	60.0 ± 2.2	45	55	487	584
Exam 15	1031	61.9 ± 2.2	45	55	460	571
Exam 16	987	63.9 ± 2.2	44	56	435	552
Exam 17	956	65.9 ± 2.2	44	56	416	540
Exam 18	893	68.1 ± 2.2	43	57	384	509
Exam 19	836	70.1 ± 2.2	42	58	351	485
Exam 20	789	72.2 ± 2.2	41	59	322	467
Exam 21	724	74.1 ± 2.2	39	61	282	442
Exam 22	646	76.0 ± 2.2	39	61	251	395
Exam 23	580	77.8 ± 2.2	37	63	217	363
Exam 24	488	79.9 ± 2.2	36	64	177	311
Exam 25	455	81.8 ± 2.2	35	65	159	296
Exam 26	376	83.8 ± 2.2	32	68	122	254
Exam 27	301	85.5 ± 2.1	31	69	94	207
Exam 28	236	87.2 ± 2.0	32	68	76	160

## Results

To understand the mortality‐predictive ability of various physiological biomarkers, we began by selecting a well‐defined and homogeneous cohort of individuals to analyze. In order to study the effect of biomarkers on mortality risk at a young age and the effects of incorporating past measurements into predictive models, we chose to focus on the youngest individuals in the original cohort of the Framingham Heart Study. The data from these individuals are both the most extensive and begin earliest in life. Moreover, this cohort, aged 28–38 years at the first Framingham exam, shares a birth decade, limiting the opportunity for confounding effects due to cultural and medical changes over time.

Of the hundreds of measurements made by the Framingham investigators, we chose to focus on basic parameters that were not indicators for any specific pathology and which had a wide dynamic range of variation (e.g., not binary variables such as whether a specific type of cardiac arrhythmia was detected on physical exam). After eliminating parameters that were not consistently measured across the first 28 clinical exams (over 50 years) of the study, we obtained a panel containing weight, height, systolic and diastolic blood pressure, BMI, and blood glucose.

### Amount of mortality predictable from physiology

**Figure 1:**
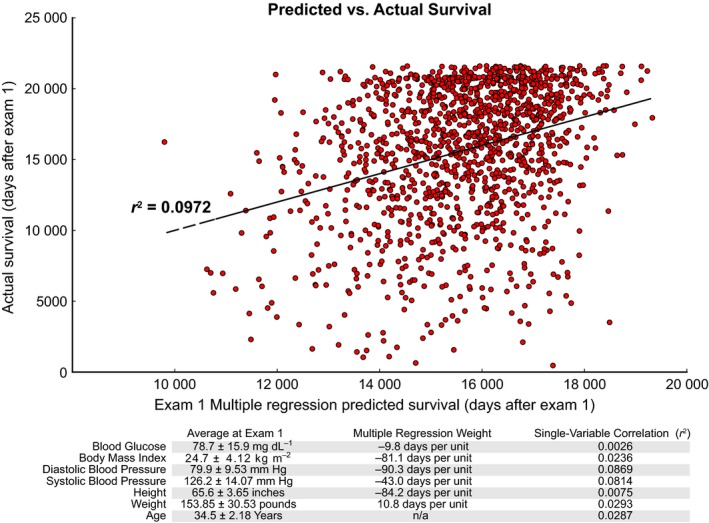
In early‐ to mid‐adulthood (participants aged 28–38), 9.7% of variation in future lifespan can be predicted from a weighted combination of systolic and diastolic blood pressure, blood glucose, height, weight, and body mass index. (top) Scatter plot of predicted lifespan from clinical measurements and actual future survival. Note that the composite predictor does not simply measure overt pathology, but has a graded response across the full dynamic range of lifespans. (bottom) Summary information for the six components of the composite predictor, along with age for comparison. At the first clinical exam, systolic pressure and diastolic blood pressure are the strongest drivers of predictions of all‐cause mortality, as shown by the *r* ^2^ for the in the single‐variable correlations with future lifespan.

In order to determine the fraction of variability in lifespan that can be predicted from the first exam, we employed multiple linear regression to assign weights to our six selected measurements. This allowed us to combine the six variables into a single composite ‘risk score’ and to assess its relationship with mortality (measured in days of survival after the first clinical exam). The relationship between predicted lifespan and results is shown in Fig. 1 (at the first clinical exam, when our cohort of participants is 28–38 years old). Overall, approximately 10% of variation in ultimate lifespan is predictable using a simple linear regression on six basic clinical parameters (*p* = 8.52 × 10^−32^).

In this type of analysis, weights for each measured parameter are chosen based on the known survival time of each individual. With sufficiently many parameters, models constructed in this fashion can become ‘overfit’ and not generalize well to future data. In this case, we have few parameter weights (seven: six measurements plus a constant offset) compared to the size of the data, and thus, the model is unlikely to be overfit. To demonstrate this directly, we estimated the ability of the model to generalize to future data using five‐fold cross‐validation, in which different subsets of the data were used to fit the weights vs. evaluate the goodness of that fit. This generated a Pearson *r* ^2^ of 0.085, suggesting that these results are indeed not overfit.

Thus, even relatively early in adulthood, a modest but meaningful fraction of future lifespan is already predictable. Further, the lack of sharp demarcations on the scatterplot indicates that this correlation is not driven exclusively by individuals with overt pathologies. Rather, the effect is graded, and is spread over much of the dynamic range that exists for both the clinical measurements and the length of known survival.

**Figure 2:**
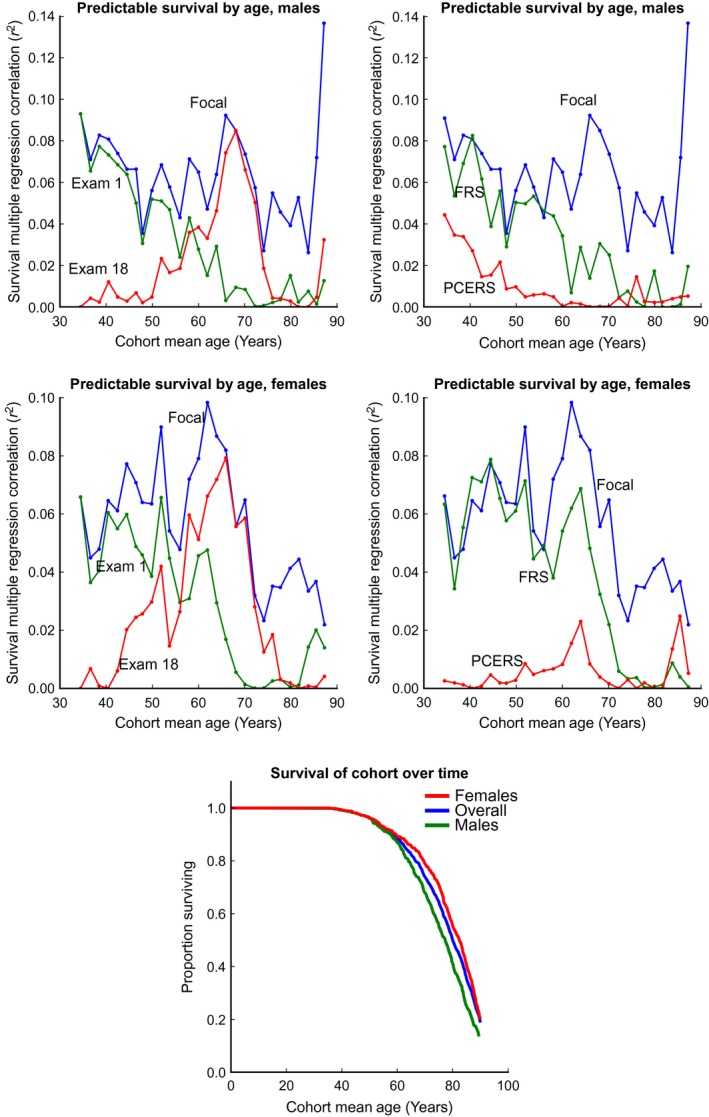
(left) The proportion of lifespan predictable from various approaches to constructing composites generally decreases with age. The trace computed by re‐calculating the regression weights at each time point independently (‘focal’) sets an upper bound on the portion of survival that predictable at each time point. The ‘exam 1’ trace performs well for early exams, but becomes less relevant at more advanced ages. Finally, the ‘exam 18’ trace, created by fitting data from individuals at clinical exam 18, performs worse than the ‘exam 1’ trace at early time points but better at later ones. (center) Our ‘focal’ exam score is compared to the Framingham Heart Score (FHS) and the Pooled Cohort Equation Risk Score (PCERS), two well‐known cardiovascular risk scores. Strikingly, the PCERS and FHS seem to behave like the ‘exam 1’ and ‘combined’ traces, respectively. Note that the FHS and PCERS are not subject to the ‘upper bound’ set by the ‘focal’ trace, as they include information from additional variables (such as blood cholesterol and age itself) not used for the ‘focal’ trace calculation. (right) A survival curve for the overall cohort studied, illustrating how the size of the surviving subcohort changes over time.

Next, we asked how the proportion of predictable mortality from single time points changes as the cohort ages. Figure 2 (left) shows the correlation between the measured parameters and mortality throughout the aging process, for the subcohort of our original population still alive at each time point, stratified for gender. (Note that the trends in these correlations thus include both the effects of age and of survivorship bias.) The three different traces represent three approaches to constructing a composite ‘risk score’ from the clinical data.

First, we used the weights that were computed to be optimal for predicting lifespan at exam 1, when the mean age of the cohort was 34.5 years (Fig. 1, bottom). The decrease in the predictive ability of this score over time suggests that the risk factors that are important in early life become less relevant in middle age. To illustrate this more explicitly, we then calculated the set of weights that most optimally predict future lifespan at exam 18, when the mean age was 68.1 years. As expected, the ‘exam 18’ trace is a worse predictor than the ‘exam 1’ trace early on, but is more effective at later time points. We next used regression to determine the optimal weighting of the six parameters at each Framingham study exam (the ‘focal exam’) independently. This provides an upper bound on fraction of lifespan predictable in a linear fashion from these basic measurements over time. (Nonlinear regression models with more free parameters may well be able to predict more about future lifespan; we excluded these from our analysis as a guard against overfitting and multiple hypothesis testing.) Of note is the gradual decline in our ability to predict mortality after roughly age 60. This suggests that the physiological parameters analyzed are able to capture relevant differences between those who die relatively early and those who die later, but not the variation among particularly longer lived individuals.

To put our findings into the context of previously developed clinical risk scores, Fig. 2 (center) compares the lifespan‐predictive ability of the well‐known Framingham Risk Score (Wilson *et al*., 1998; Lloyd‐Jones *et al*., 2004) and Pooled Cohort Score (Goff *et al*., 2014) with our ‘focal exam’ estimates. These clinical scores were designed specifically to assess cardiovascular risk in a 5‐ to 10‐year timeframe, using some of the parameters we examined as well as smoking status, blood cholesterol, and age. Despite this narrow focus, however, both scores are able to predict a significant proportion of variation in all‐cause mortality over 50+ years of follow‐up. Indeed, their predictive value remains valid even at the earliest clinical exam, when the average age of participants is 34.5 years. This demonstrates a surprising robustness of the lifespan‐predictive signal in these data: even risk scores such as these, which were not specifically tuned to the task, are effective biomarkers of aging.

**Figure 3:**
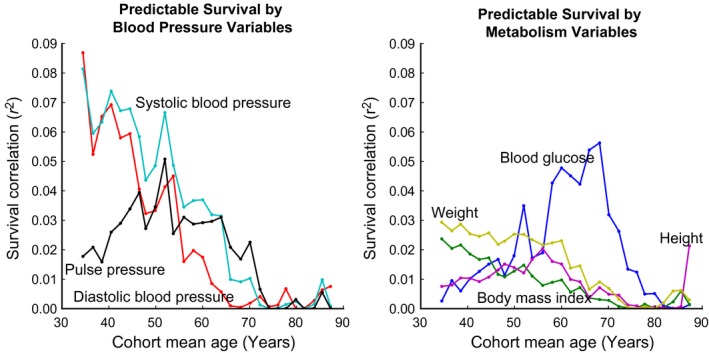
The individual variables' ability to predict survival changes over time. Blood pressure, BMI, and weight are predictive of mortality primarily from ages 35 to 60 and while blood glucose is most predictive from ages 57 to 73. Also, note that BMI, all three measures of blood pressure, and weight all decrease in predictivity as the cohort ages, but blood glucose's ability to predict survival increases in middle age before declining again.

Next, Fig. 3 illustrates how the individual measurements' (from Fig. 1) correlations with future lifespan change over time. As expected, the predictive ability of most of the parameters under investigation declines as the overall predictable mortality (measured by the focal exam trace from Fig. 2) declines. This trend is most strongly illustrated by the three blood pressure variables, systolic, diastolic and pulse pressure, which are highly predictive of lifespan from ages 35 to 60 and increasingly less predictive thereafter. On the other hand, blood glucose is a striking exception. Up to age 70, as all other significant predictors' correlations are declining, the correlation of blood glucose with all‐cause mortality rises consistently, achieving its highest values from the ages of 57–73. Overall, it appears that blood pressure and BMI are predictive of all‐cause mortality early in life (ages 35–60, with pulse pressure interestingly being a stronger predictor in late life than systolic and diastolic blood pressure), while blood glucose is more meaningful in middle age (ages 57–73). This reinforces the evidence from Fig. 2 that the mortality‐predictive ability of our clinical variables changes during different phases of life.

### Incorporating physiological history into mortality prediction

**Figure 4:**
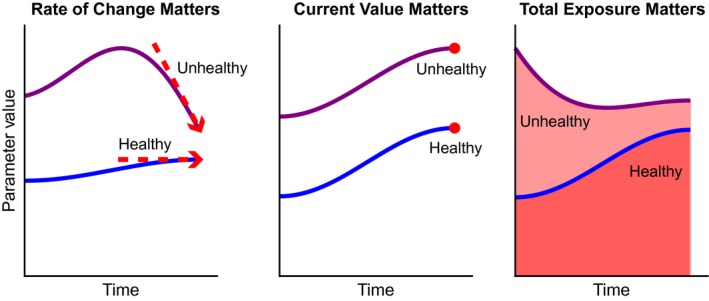
Hypotheses for the relationship between health (risk of mortality) and trends in physiological measurements. The potential scenarios considered are as follows: (left) the derivative (‘rate of change’) of a measurement is the most relevant quantity for predicting mortality; (center) the current value of the measured parameter is most predictive of mortality; (right) the integral (‘accumulated exposure’) is most predictive of mortality. In each panel, a blue line represents a ‘healthy’ time course, while a purple line represents a more ‘unhealthy’ time course at higher risk for mortality. In the left panel, the precise values of the measurements over time are not important, but their stability over time is. Thus, a trajectory in the midst of a sharp decline may be a risk factor, while a more stable trajectory is lower risk. The middle panel illustrates the case in which a parameter value directly relates to underlying risk, so the current value of that parameter is most useful as a quantitative risk factor. Finally, the right panel shows the case when an individual's accumulated exposure is the relevant risk factor. Here, the total historical exposure (area under the curve) is more predictive of mortality than the measure's current value or rate of change; as such, in this example, the trajectory with the larger shaded area underneath is the more at‐risk.

As the Framingham data provide measurements of the same physiological parameters at different ages for each individual, we next investigated whether and how trends in these measures over time are predictive of future lifespan (Fig. 4). The simplest possibility is that a particular measurement directly reflects the current state of health of an individual at that moment (central panel in Fig. 4). In this case, the previous values of that measurement are redundant/irrelevant. Alternately, there may be cases where the precise value of a clinical measurement itself is not particularly important for prognosis, but its rate of change with time is more reflective of future health (illustrated by the slope of the tangent line in the left panel in Fig. 4). For example, progressive increases in the size of a skin blemish could indicate a malignancy, while the actual size of the blemish itself may be less relevant. Finally, other clinical measures may directly capture rates of change of health, such that the relevant information for prognosis is the cumulative change over time (the integrated ‘area under the curve’, illustrated by the shaded region in the right panel in Fig. 4). One clear example is an individual's risk from their history of smoking. In that case, there is significant evidence that one's smoking ‘rate’ (packs per day) is in some sense a rate of decline of health and that the true risk is best predicted by total accumulated underlying damage (typically reported as ‘pack‐years’ of smoking), rather than the current smoking rate.

To see which of these possibilities best fits the observed data, we computed ‘rates of change’ (i.e., differentiated) and ‘cumulative’ (i.e., integrated) versions of our variables and analyzed their correlations with mortality. We first attempted several formulations of ‘rates of change’: taking the difference between successive exams; reducing noise by finding the slope of a line fitted to three exam time points rather than two; and smoothing the data by averaging multiple adjacent exams before obtaining pointwise rates of change as above. We found that no ‘rate of change’ formulation was predictive of mortality (data not shown).

We next focused on the accumulation hypothesis, in which integrating past values for a given measurement might yield improved predictive ability. To explore the idea of accumulated risk, we constructed variables analogous to pack‐years for smoking by simply summing past measurements, weighed to account for the uneven timing of the Framingham exams. If, as hypothesized, a particular variable represents a ‘rate of change’ of health, then its accumulated history will be more predictive of mortality than its current single time value. Continuing the example of smoking, an individual's accumulated pack‐years of smoking should have a stronger correlation with survival than their current smoking rate. Further, looking at intermediate amounts of history should yield intermediate levels of predictive capacity. For example, if we obtain individuals' smoking history for only the previous 5 years, that variable's correlation with mortality should be greater than the correlation from the single time measurement, but less than the correlation obtained for their full lifetime history of smoking. If, on the other hand, integrating the most recent 5 years or so of history improves predictive abilities but earlier historical data adds nothing, this may suggest that the ‘integrated recent history’ may simply be a de‐noised estimate of the current physiological state. While such denoising may be clinically useful, it is not good evidence that the risk factor truly is cumulative.

**Figure 5:**
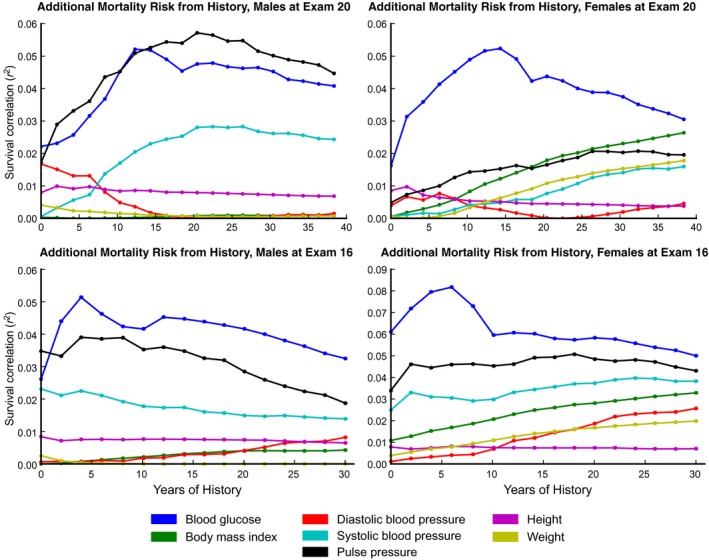
A plot of how the mortality‐predictive ability of single variables changes as more history is incorporated into an accumulated risk score at ‘focal exams’ 16 and 20. Accumulating variables are identified by a steady increase in mortality‐prediction with each additional unit of history. At exam 16, diastolic blood pressure exhibits a consistent increase in mortality‐prediction with each additional unit of history for both men and women, although the effect is smaller in size for men. In contrast, at exam 20, blood glucose accumulates predictivity with additional history for approximately 15 years, corresponding to ages 57–73. BMI, on the other hand, behaves as an accumulating variable for women at focal exams 16 and 20, but is relatively unpredictive of mortality for men at these exams.

**Figure 6:**
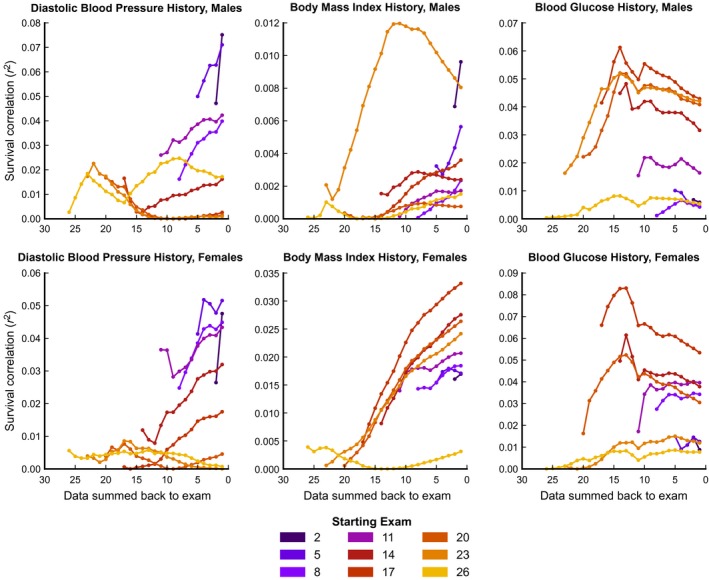
The accumulation of predictivity using additional years of history, starting at different exams. Each single time trace shown here is analogous to a single time trace in Fig. 5. Each panel illustrates how additional years of historical data improve risk prediction for a different physiological variable. Unlike Fig. 5, which presents these results from exams 16 and 20, here a range of ‘focal exams’ are shown. Each trace represents the change in mortality predictivity as additional years of history are incorporated into the mortality prediction from a given starting point (‘focal exam’). For example, the yellow traces show how the predictive ability of a given measure at exam 26 can be increased by adding additional historical data. Each particular point on a yellow trace then represents the gain in predictive ability when data area (as illustrated in the right panel of Fig. 4) is added from exam 26 back to that particular point in time. As in Fig. 5, the hallmark of an accumulating variable is a steady increase of predictivity with the incorporation of additional history. Note that BMI seems to accumulate predictivity as more history is incorporated, regardless of the focal exam from which the analysis is begun. In contrast, diastolic blood pressure and blood glucose only accumulate predictivity in limited regimes. Diastolic blood pressure and blood glucose accumulate risk from exams 1 to 14 and 13 to 20, respectively, which correspond to average ages of 35–60 and 57–73 years.

For this analysis, we begin with the single time correlation at a particular exam (the ‘focal exam’), then trace the mortality‐predictive abilities of individual clinical parameters as progressively more years of history are incorporated (Fig. 5). In this Figure, each successive point in the line includes history from one additional Framingham study exam. As more history is incorporated (by summing variable‐years), some variables' correlation with survival increases consistently and roughly linearly, suggesting that these variables are indeed cumulative. Figure 5 shows these traces with exams 16 and 20 as the focal exams. (These exams were conducted when the participants were 59–69 and 67–77 years old, respectively.) For focal exam 16, diastolic blood pressure exhibits a consistent increase in mortality‐prediction with each additional unit of history for both men and women. In contrast, for focal exam 20, diastolic blood pressure does not seem to accumulate predictivity from history, while blood glucose does. We have also excluded the effects of hypertension treatment in Figs 5 and 6. This caused the size of the observed effects to decrease marginally without creating any substantial differences from the same analysis of data uncontrolled for hypertension treatment (data not shown). We believe that this steady increase in mortality‐predictive capacity with additional historical data is the hallmark of a variable that affects health through cumulative exposure. The switch in which variables predict future risk at different ages between exams 16 and 20 is consistent with our earlier analysis, and much other work (Albala *et al*., 1996) demonstrating the dramatic change in risk factors with age (Fig. 3).

To further examine these trends, we focused on diastolic blood pressure, BMI and blood glucose, which appear to be cumulative when considered from the perspective of exams 16 and 20. For each variable, Fig. 6 shows the utility of additional years of history when starting from a range of focal exams, rather than just exams 16 and 20. In particular, BMI seems to increase in mortality‐predictive capacity as more history is incorporated, regardless of the clinical exam from which we begin the analysis. In contrast, diastolic blood pressure appears to stop showing signs of cumulative risk after clinical exam 17, which corresponds to an average age of 65.9 years. Blood glucose, however, seems to behave as an accumulating variable for all exams after clinical exam 13, which corresponds to an average age of 58.0 years. Again, this extends our result from earlier: Not only do blood glucose and blood pressure seem to be important for mortality‐risk prediction at different age regimes (ages 35–60 and 57–73, respectively), their predictive capability within their regimes is cumulative (ages 35–65, and 58–87, respectively); that is, blood glucose and blood pressure appear to primarily predict mortality through accumulated lifetime exposure.

## Discussion

We surveyed the amount of variation in all‐cause mortality that could be predicted from physiology at early‐ to mid‐adulthood, finding a modest but nontrivial effect: 9.7% of variation in mortality at ages 28–38 can be predicted using the common physiological measurements of height, weight, BMI, blood glucose level, and systolic and diastolic blood pressure. In addition, we have shown that our results are generally valid for both men and women. Although there are several quantitative differences in the magnitudes of effects and their precise timing, our overarching conclusions apply to both genders.

Further, the amount of predictable mortality generally decreases as a cohort ages and the least healthy individuals die off. This result is consistent with survivorship bias: as the unhealthy individuals die off, the surviving cohort becomes more homogeneous. As a result, the surviving cohort will have less predictable variation in survival; that is, a greater proportion of the variation will be random and not predictable.

Specifically, Fig. 3 shows that the ability of blood pressure (diastolic, systolic, and pulse pressure), BMI, and weight to predict variation in survival is high in early‐ to mid‐adulthood, and gradually decreases up to roughly age 60. Although there is some fluctuation in this trend, likely the result of measurement noise, the overall direction for most variables is clear. Blood glucose's predictivity presents an interesting exception. While essentially all other variables are decreasing in predictive capacity (up to age 60), blood glucose's predictive ability seems to sharply increase from age 57, peaking around the age of 70. The fact that blood pressure, BMI, and weight are predictive of mortality primarily from ages 35 to 60 and while blood glucose is most predictive from ages 57 to 73 suggests a fundamental difference between their contributions to all‐cause mortality.

In parallel, Fig. 6 demonstrates that harm mediated by hypertension and obesity accumulates from ages 35 to 65, while mortality risk from blood glucose accumulates from ages 58 to 87. In conjunction with the results from Fig. 3 discussed above, these results suggest certain ‘critical periods’ (Dietz, 1994) for damage accumulation and mortality predictivity. These ‘critical periods’ vary between different physiological parameters, but seem to represent times of damage accumulation and high mortality predictivity for each parameter. Again, all‐cause mortality risk before age 70 seems is best predicted using blood pressure and BMI, while mortality during the age regime from age 60 and through age 80 is associated with blood glucose. While the confounding effects of changing societal habits over the years need to be taken into account, this type of analysis promises to yield important insights into the evolution of health and accumulated mortality risk over the course of the aging process.

The simplistic explanation for these observed ‘critical periods’ is that the changing predictive ability of blood glucose and other measurements is entirely due to the aging process: In general, perhaps blood pressure becomes a less important predictor with increasing age while the predictive value of blood glucose increases. This interpretation, however, is confounded by the changing life habits of the American population over recent history. Shifts in diet and lifestyle have steadily increased the contribution of diabetes mellitus to morbidity and mortality over the past half‐century, and advances in hypertension treatment have altered the physiological interpretation of blood pressure measurements. Disentangling these effects calls for additional analysis of both the Framingham data as well as other cohorts.

In addition to surveying mortality predictivity in the single‐time‐point context, we investigated trends in variables over time. Rates of change for height, weight, BMI, blood glucose level, and systolic and diastolic blood pressure were utterly unpredictive of future mortality, despite several different approaches to estimating these rates of change. However, in many cases the measured variables' accumulated history yielded substantially higher correlations in comparison with the single‐time‐point context.

Specifically, the ability of diastolic blood pressure and BMI to predict all‐cause mortality risk increases steadily and roughly linearly as more historical information is included for individuals below the age of 67. For blood glucose, this effect is observed most prominently from the age 58 onward. In conjunction with our result that indicates that blood glucose is mortality‐predictive at later ages than blood pressure and BMI, these ‘accumulating regimes’ indicate that these variables' effects on mortality are cumulative.

This is consistent with the current understanding of the harms mediated by obesity, high blood glucose, and high blood pressure. While the precise mechanisms by which high blood pressure is pathological are still a subject of active research, the consensus so far is that the hemodynamic forces of hypertension initiate a signal which is transduced by endothelial cells, initiating various pathways involving ion channels, growth factors, extracellular matrix interactions, and various other molecular components (Luft *et al*., 1999). The end result of these effects is generally a combination of vascular remodeling – blood vessels becoming less pliable, weaker, narrower – and specific organ damage, commonly in kidneys. Overall, it is generally agreed that the harmful effects of hypertension occur gradually over time. Similar lines of evidence suggest that pathologies from high blood glucose and obesity occur gradually as well (Kahn *et al*., 2006).

Thus, it is not merely having high blood pressure, high blood glucose, or being overweight that instantaneously puts one at risk for the various associated pathologies. Rather, it is the sustained level of increased stress on one's organ systems that causes a gradual accumulation of damage. This suggests that many current preventative medicine guidelines, which focus on cardiovascular risk factors in older patients, may be failing to fully capture the lasting dangers of an unhealthy lifestyle, especially for younger individuals. In addition, while the concept of additional risk from sustained lifetime exposure may be intuitively understood by physicians, this study quantitatively highlights the magnitude of the effect, and suggests a straightforward way to estimate risk from accumulated exposure: simply summing historical physiological measurements. Further, some recent work on functional impairment during the aging process, as measured by frailty indices (Kulminski *et al*., 2007; Mitnitski *et al*., 2013), may be evidence of downstream effects of accumulated physiological damage.

With the advent of electronic medical records and ever‐increasing computing power, it is becoming commonplace to have access to patients' previous physiological measurements. By exploiting longitudinal information in novel ways, we have shown that the predictive capacity of many well‐studied risk factors can be greatly increased. Further, these statistical improvements can also drive new biomedical insights and provide avenues for testing those hypotheses. Much previous work has demonstrated the utility of physiological history for understanding the processes that drive aging and the relationship between disease risk and specific physiological measurements (Yashin *et al*., 2006, 2007; Arbeev *et al*., 2011). Our results add to this understanding by demonstrating clearly that early‐life physiology informs late‐life survival and that cumulative historical exposure can be a useful variable to track in addition to the present state of an individual's physiology. Incorporating early‐life data (where available) and recent history into existing quantitative models may further improve our understanding of how and why mortality risk increases with aging.

## Experimental procedures

### Data collection

The design of the Framingham Heart Study has been previously described (Kannel *et al*., 1979; Collins *et al*., 1990). For our present study, subjects in the original cohort who were ages 28 to 38 at the first examination (1948–1953, 1451/5079 subjects) were eligible. We excluded participants that were lost to follow‐up (defined by lacking both a known date of death and having no recorded data from clinical exam 28) and those who were missing sufficient data for any of the six physiological measures we studied (systolic blood pressure, diastolic blood pressure, blood glucose, height, weight, BMI). After applying our criteria, 91 individuals were excluded on the basis of incomplete follow‐up and 10 individuals were excluded on the basis of insufficient data. We then manually removed a single outlier individual, who had a systolic blood pressure of 250 mm Hg at the first clinical exam (an extraordinarily pathological reading, approximately 8.8 standard deviations from the mean) and died approximately 3 months later. We thus obtained a cohort of 1349 participants who remained eligible for this study. In Table 2, we see that the differences between our chosen cohort and individuals excluded for lack of follow‐up or insufficient data are minimal, and are unlikely to cause significant bias in our results. By selecting a birth decade cohort, we hope to minimize the confounding effects of age, as all participants in our study are similar in age.

**Table 2:** Comparison of the selected vs. excluded Framingham Heart Study (FHS) participants

	Average at Exam 01, selected males	Average at Exam 01, excluded males	*P*‐value (*t*‐test), males	Average at Exam 01, selected females	Average at Exam 01, excluded females	*P*‐value (*t*‐test), females
Blood glucose	79.1 ± 18.65 mg dL^−1^	77.1 ± 13.11 mg dL^−1^	0.57	78.3 ± 12.86 mg dL^−1^	76.8 ± 10.42 mg dL^−1^	0.39
Body mass index	25.6 ± 3.71 kg m^−2^	24.0 ± 3.0 kg m^−2^	0.02	23.8 ± 4.28 kg m^−2^	22.6 ± 4.16 kg m^−2^	0.03
Diastolic blood pressure	82.7 ± 9.77 mm Hg	83.5 ± 13.38 mm Hg	0.69	77.4 ± 8.56 mm Hg	76.0 ± 7.8 mm Hg	0.2
Systolic blood pressure	131.1 ± 14.04 mm Hg	133.1 ± 24.38 mm Hg	0.47	121.9 ± 12.59 mm Hg	119.4 ± 11.7 mm Hg	0.11
Height	68.4 ± 2.74 inches	68.3 ± 2.07 inches	0.82	63.1 ± 2.34 inches	63.3 ± 2.63 inches	0.6
Weight	172.58 ± 26.24 pounds	161.83 ± 21.31 pounds	0.03	137.34 ± 23.78 pounds	132.0 ± 23.3 pounds	0.07
Age	34.5 ± 2.16 years	33.9 ± 2.13 years	0.18	34.5 ± 2.19 years	34.0 ± 2.2 years	0.08
Number of participants	631	30		718	72	

All parameter mean values are within a single standard deviation, suggesting that our exclusion criteria did not introduce any obvious bias. The only statistically significant differences (at a *P* = 0.05 level) between included and excluded cohorts are in BMI and weight. However, even for BMI and weight, the excluded individuals' mean values are still within a standard deviation of those of the included cohort. Further, relatively few individuals were excluded in relation to those who remained in the cohort, so we believe the risk of bias from our exclusion criteria to be minimal.

### Statistics

Statistics (Pearson correlation coefficient, multiple regression weights, partial correlations) were computed using the Python programming language and associated packages.

### Construction of clinical risk scores

It should be noted that although we made every effort to reconstruct the Framingham and Pooled Cohort Equations risk scores as completely as possible, the smoking and high‐density lipoprotein cholesterol terms had to be excluded due to the lack of consistent longitudinal measurement in the Framingham Heart Study. For example, early blood cholesterol measurements did not distinguish between high‐, low‐, and very low‐density lipoproteins. The data from smoking questionnaires also varied among clinical exams, and were difficult to interpret consistently for all longitudinal time points.

### Blood pressure treatment

To minimize the confounding effects of blood pressure treatment in Figs 5 and 6, we adjusted individuals' blood pressure measurements to reflect our best estimates of their untreated blood pressure. We achieved this by adding the average effect of blood pressure treatment to all individuals. Our results computed with controlled blood pressure were not substantially different from those from the same analysis on uncontrolled blood pressure (data not shown).

### Pulse pressure

Pulse pressure, a derived variable defined as the difference between an individual's systolic and diastolic blood pressures, was included as a seventh variable in our analysis from Fig. 3 onward. We excluded pulse pressure from Figs 1 and 2, which employed multiple linear regression, because it is linearly dependent on systolic and diastolic blood pressure and would not have made any meaningful contribution to the derived composite variable.

### Exam weighting in history accumulation

To adjust for unequal timing of clinical exams, we assumed that at any given point in time, each patient has the same measurement as the closest clinical exam. For example, if a patient has a measured systolic blood pressure (in mm Hg) of 120 in 1960, 130 in 1966, and 140 in 1976, our analysis would use this adjustment to assign blood pressures of 120 for 1960–1963, 130 for 1964–1971, and 140 for 1972–1976. Further, to avoid artificially diminishing the impact of the first and last available time points, we pad the first and last exams with half of the average interexam period. In the given example, we would actually assign blood pressures of 120 for 1956–1963, 130 for 1964–1971, and 140 for 1972–1980.

## Funding

ZP and WZ are supported by NIH grant R00 AG042487 and Longer Life Foundation grant 2015‐008. WZ is additionally supported by NIH grant 5T32 GM07200.

## Conflict of interest

None declared.

## Author contributions

WZ and ZP designed the experiments. WZ carried out the analysis, and WZ and ZP wrote the manuscript.

## Supporting information


**Fig. S1.** For men in early‐to‐mid adulthood (participants aged 28–38), 9.3% of variation in future lifespan can be predicted from a weighted combination of systolic and diastolic blood pressure, blood glucose, height, weight, and body mass index.
**Fig. S2.** For women in early‐to‐mid adulthood (participants aged 28–38), 6.6% of variation in future lifespan can be predicted from a weighted combination of systolic and diastolic blood pressure, blood glucose, height, weight, and body mass index.
**Fig. S3.** The individual variables' ability to predict survival in a single‐time point context, stratified by gender.
