# Supplementary material for: Predicting all‐cause mortality from basic physiology in the Framingham Heart Study
Source: Aging Cell. 2015 Oct 8;15(1):39–48. doi: 10.1111/acel.12408 (PMC4717277; doi:10.1111/acel.12408)
Supplement: Supplementary file 1 — Fig. S1. For men in early‐to‐mid adulthood (participants aged 28–38), 9.3% of variation in future lifespan can be predicted from a weighted combination of systolic and diastolic blood pressure, blood glucose, height, weight, and body mass index. Fig. S2. For women in early‐to‐mid adulthood (participants aged 28–38), 6.6% of variation in future lifespan can be predicted from a weighted combination of systolic and diastolic blood pressure, blood glucose, height, weight, and body mass index. Fig. S3. The individual variables' ability to predict survival in a single‐time point context, stratified by gender. [file ACEL-15-039-s001.pdf]

Supplementary Information

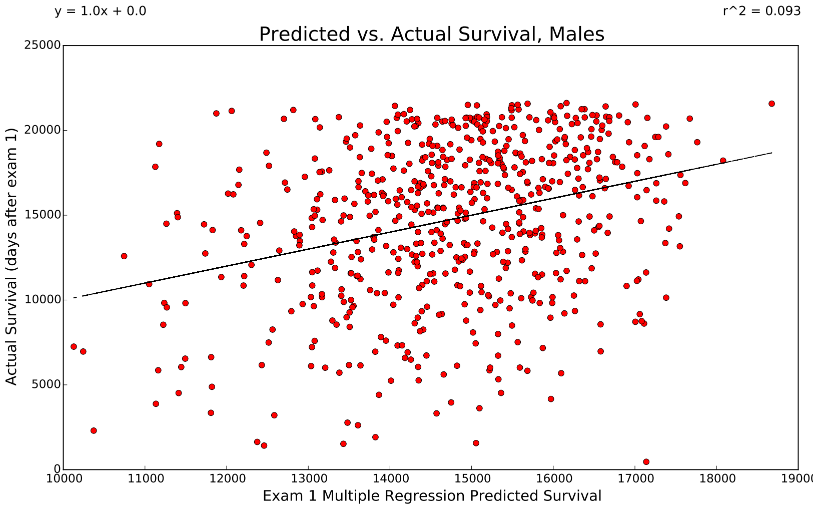

	Average at Exam 01	Multiple Regression Weight	Single-Variable Correlation r^2
Blood Glucose	79.1 ± 18.65 mg/dL	-11.2 days per unit	0.0029
Body Mass Index	25.6 ± 3.71 kg/m^2	-394.0 days per unit	0.0126
Diastolic Blood Pressure	82.7 ± 9.77 mm Hg	-94.9 days per unit	0.0792
Systolic Blood Pressure	131.1 ± 14.04 mm Hg	-41.9 days per unit	0.0671
Height	68.4 ± 2.74 inches	-225.0 days per unit	0.0060
Weight	172.58 ± 26.24 pounds	65.44 days per unit	0.0038
Age	34.5 ± 2.16 years	nan	0.0247

Figure S1: *For men in early-to-mid adulthood (participants aged 28–38), 9.3% of variation in future lifespan can be predicted from a weighted combination of systolic and diastolic blood pressure, blood glucose, height, weight, and body mass index. This is marginally less than the predictable portion of variation for men and women combined. This indicates that our composite predictor is to some extent capturing male-female differences in physiology and their effect on lifespan.*

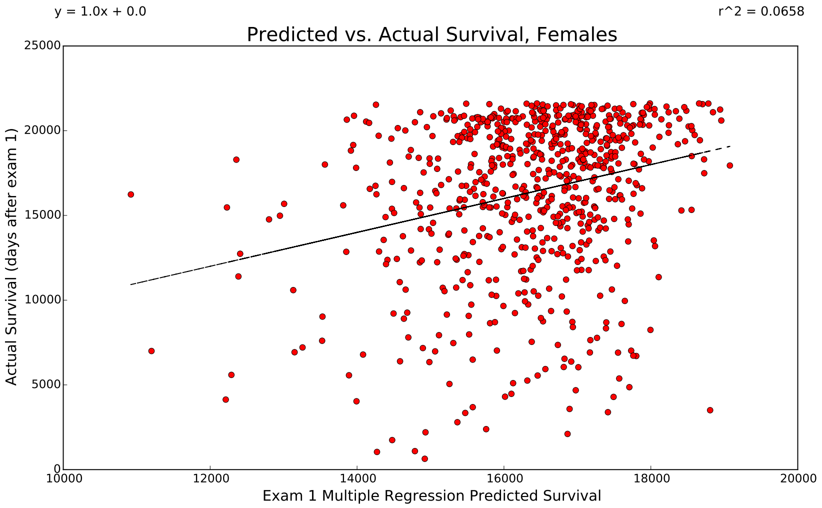

	Average at Exam 01	Multiple Regression Weight	Single-Variable Correlation r^2
Blood Glucose	78.3 ± 12.86 mg/dL	-9.2 days per unit	0.0020
Body Mass Index	23.8 ± 4.28 kg/m^2	239.4 days per unit	0.0167
Diastolic Blood Pressure	77.4 ± 8.56 mm Hg	-77.5 days per unit	0.0580
Systolic Blood Pressure	121.9 ± 12.59 mm Hg	-39.9 days per unit	0.0546
Height	63.1 ± 2.34 inches	241.9 days per unit	0.0007
Weight	137.34 ± 23.78 pounds	-48.48 days per unit	0.0141
Age	34.5 ± 2.19 years	nan	0.0338

Figure S2: *For women in early-to-mid adulthood (participants aged 28–38), 6.6% of variation in future lifespan can be predicted from a weighted combination of systolic and diastolic blood pressure, blood glucose, height, weight, and body mass index. This is less than the predictable portion of variation for men and women combined. This indicates that our composite predictor is to some extent capturing male-female differences in physiology and their effect on lifespan. Another possible contributing factor is that more women remain alive at the latest clinical exam, so follow-up is less complete for women than for men.*

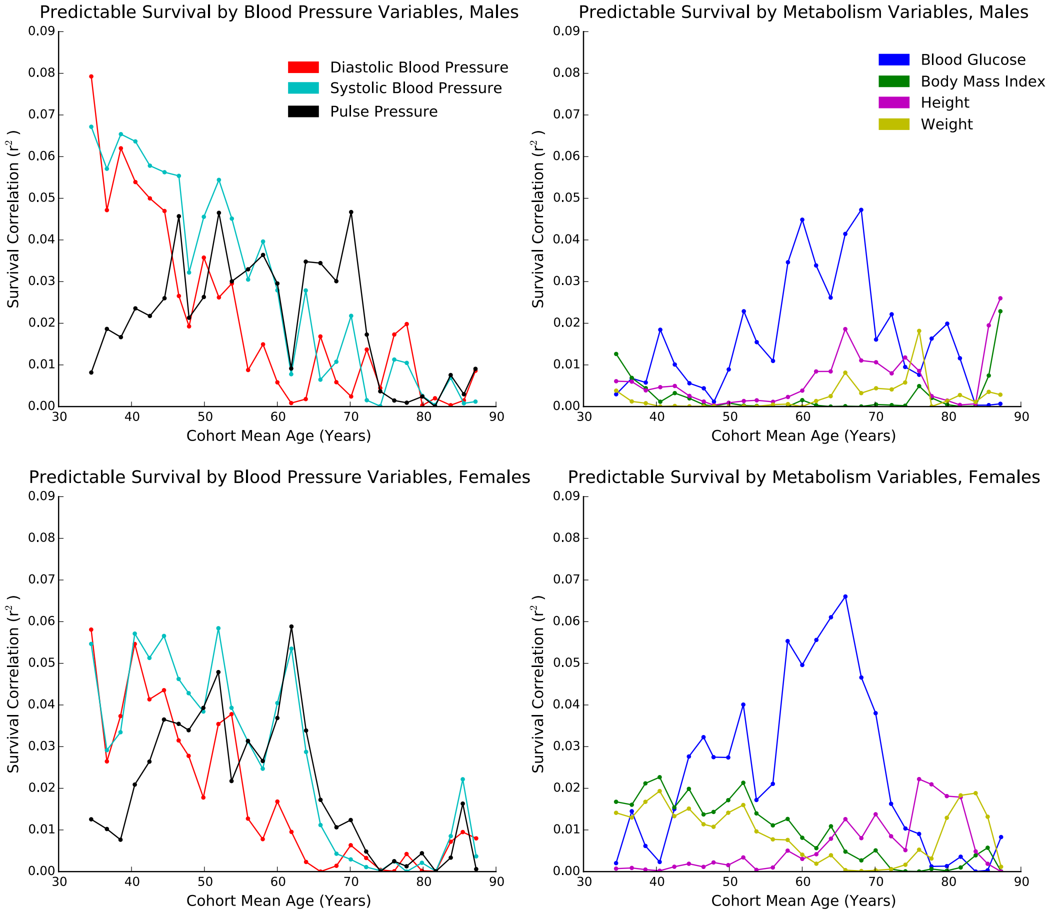

Figure S3: The individual variables' ability to predict survival in a single-time point context, stratified by gender. We see that the overall patterns of mortality-predictivity are very similar for women and men, though relatively minor differences in the magnitude of predictable mortality and the timing oflongitudinal trends exist. As in figure 3 in the main text, blood pressure, BMI, and weight are predictive of mortality primarily from ages 35-60 and while blood glucose is most predictive from ages 57–73.
